# Deep learning classification of reading disability with regional brain volume features

**DOI:** 10.1016/j.neuroimage.2023.120075

**Published:** 2023-04-11

**Authors:** Foram Joshi, James Z. Wang, Kenneth I. Vaden, Mark A. Eckert

**Affiliations:** a School of Computing, Clemson University, Clemson, S.C, U.S.A; b Department of Otolaryngology - Head and Neck Surgery, Medical University of South Carolina, Charleston, S.C, U.S.A

**Keywords:** Specific learning disorder in reading, Dyslexia, Reading disability, Brain morphology, Convolutional neural network

## Abstract

Developmental reading disability is a prevalent and often enduring problem with varied mechanisms that contribute to its phenotypic heterogeneity. This mechanistic and phenotypic variation, as well as relatively modest sample sizes, may have limited the development of accurate neuroimaging-based classifiers for reading disability, including because of the large feature space of neuroimaging datasets. An unsupervised learning model was used to reduce deformation-based data to a lower-dimensional manifold and then supervised learning models were used to classify these latent representations in a dataset of 96 reading disability cases and 96 controls (mean age: 9.86 ± 1.56 years). A combined unsupervised autoencoder and supervised convolutional neural network approach provided an effective classification of cases and controls (accuracy: 77%; precision: 0.75; recall: 0.78) Brain regions that contributed to this classification accuracy were identified by adding noise to the voxel-leve image data, which showed that reading disability classification accuracy was most influenced by the superior temporal sulcus, dorsal cingulate, and lateral occipital cortex. Regions that were most important for the accurate classification of controls included the supramarginal gyrus, orbitofrontal, and medial occipital cortex. The contribution of these regions reflected individual differences in reading-related abilities, such as non-word decoding or verbal comprehension. Together, the results demonstrate an optimal deep learning solution for classification using neuroimaging data. In contrast with standard mass-univariate test results, results from the deep learning model also provided evidence for regions that may be specifically affected in reading disability cases.

## 1. Introduction

Specific learning disability of reading, or reading disability, is a complex disorder that occurs in approximately 5 – 17% of the population (Grigorenko et al., 2020; Moll et al., 2014; Rumelhart et al., 1985; Shaywitz et al., 1990), depending on how reading disability is defined (Peterson and Pennington, 2015), and is often characterized by difficulty in the accurate and/or fluent recognition of words (Fletcher, 2009). These difficulties reflect problems with phonological aspects of language, which can occur independently of ability for other cognitive abilities and quality of classroom instruction (Lyon et al., 2003). Reading disability can occur with other learning disabilities where oral language, comprehension, and written expression skills are affected (Berninger and Abbot, 2010; Bishop and Snowling, 2004; Moll et al., 2014). Many children with reading disability experience secondary consequences that include problems in reading comprehension and limited reading experience, which slow the growth of vocabulary and general knowledge (Cain and Oakhill, 2011; Duff et al., 2015). Some children with reading comprehension problems, however, do not have the classic phonological impairments of children with reading disability (Catts et al., 2012; Cutting et al., 2013; Locascio et al., 2010; Peterson and Pennington, 2015). While there is structural imaging evidence of consistently affected brain regions, such as the superior temporal sulcus, based on meta-analysis findings (Eckert et al., 2016; Richlan et al., 2013), effect sizes in structural neuroimaging studies are often varied and are not always replicated (Ramus et al., 2018). This replication problem may be due to the behavioral heterogeneity of reading disability samples, small sample sizes, and false positives due to the large number of variables that are often examined in voxel-based studies (Moreau et al., 2018).

The behavioral and neural heterogeneity of reading disability samples may explain why machine learning classifiers of dyslexia can be effective within carefully selected small samples, which nonetheless may not generalize well. For example, a measure of real word reading was related to support vector machine (SVM) classification scores that were based on a large number of white matter variables, which included measures from the fornix, left cingulum, and left superior frontal-occipital fasciculus in a relatively small sample of 28 dyslexia cases and 33 controls that were classified with 84% accuracy (Cui et al., 2016). While a positive result, the degree to which these results would generalize should be considered in the context of another small sample study where SVM was performed using gray matter volume features selected from voxels exhibiting the largest differences between 22 control and 27 dyslexic adults, which included the bilateral fusiform gyri and left inferior parietal lobule (Tamboer et al., 2016). Although there was 80% training accuracy within the training sample, accuracy decreased to 59% when the classifier was tested in an independent sample of adults. Sample heterogeneity may also have affected traditional classification measures, such as SVM, random forest (RF), and logistic regression using brain morphology features, which included sulcal/gyral shape measures of the left middle temporal gyrus, left planum polare, and frontopolar cortex in a multi-site dataset given a modest ~65% accuracy in 236 participants (Płoński et al., 2017). Deep learning approaches using stratification by research site can limit the impact of this heterogeneity, and perhaps varied image acquisition across sites, on classification.

Classification with neuroimaging data is often described as being affected by the “curse of dimensionality”, or the high number of measurements (i.e., voxels) that can lead models to over-fit to a small number of participants during training and then fail to generalize learning to other datasets. For this reason, feature selection approaches have been used [e.g., features based on univariate *t*-test comparisons between groups used for classification (Cui et al., 2016)], although these approaches also can lead to poor generalization. In the current study, autoencoders were used to reduce the dimensionality of structural imaging datasets collected from eight different research sites. This analysis included a proportional selection approach to deal with potential research site differences in sampling and image acquisition.

The current study included an unsupervised learning approach that extracted relevant latent representations of voxel-based brain morphology features before using them in the supervised classification. A stacked autoencoder (AE) for feature extraction and data compression was first implemented, followed by a 3D-convolutional neural network (CNN) (Krizhevsky et al., 2012) architecture that predicted whether the latent representation of a brain image, or summary of key features in the image, was consistent with a reading disability or control classification. This latent representation of the image addressed the curse of dimensionality, as discussed earlier. After establishing the classification approach, analyses were performed to identify brain regions that were most important for accurate classification using an image perturbation approach, and whether these results replicated findings from previous imaging studies of dyslexia (Krishnan et al., 2016; Richlan et al., 2013), including subcortical regions linked to reading disability (Eckert et al., 2017; Krishnan et al., 2016). A key advantage of this approach was the opportunity to determine whether specific regions were critical for reading disability and/or control classification.

This study was designed to evaluate and advance deep learning methods for predicting reading disability, with the potential for application to other disorders or diseases. We hypothesized that brain regions observed to exhibit consistently atypical morphology in reading disability studies (e.g., the left superior temporal sulcus in the meta-analyis studies by Eckert et al., 2016; Richlan et al., 2013), as well as brain regions considered to be part of a reading network (e.g., left inferior parietal lobule, left occipitotemporal regions: Paulesu et al., 2014; Zang and Peng, 2022) would exhibit lower volume in reading disability cases compared to controls and would have relatively greater importance for reading group classification accuracy than other brain regions. Because specific learning disability of reading is considered a dimensional disorder, we also examined the extent to which poorer reading skills occurred with lower volume within brain regions that with relatively greater importance for reading group classification accuracy.

## 2. Materials and methods

### 2.1. Participants

Data used in the current study were contributed by members of the Dyslexia Data Consortium (www.dyslexiadata.org) and collected as part of studies of reading disability group differences and dimensional studies of reading development. Data from a subset of the children in this study have been reported previously (Eckert et al., 2016, 2020, 2017) and represent eight different research sites, consisting of 96 cases with reading disability (38 females; age $\overset{-}{x}=9.93\pm 1.55$) and 96 controls (38 males; age $\overset{-}{x}=9.79\pm 1.57$). These data were collected at the contributing institutions with informed consent and approval from each institution’s Internal Review Board (IRB). Approvals were also obtained at each contributing site to share the data and the Medical University of South Carolina IRB provided approval to receive the de-identified data.

### 2.2. Reading disability groups and behavioral assessment

The reading disability cases and controls were defined using a supervised reading disability classifier (Eckert et al., 2017). This classifier was trained using labels from two clinically certified Speech-Language Pathologists who had grouped children into different reading disability profiles based on non-word reading, real word reading, passage comprehension, verbal comprehension, and rapid naming measures that are often used clinically to assess for reading disability. Specifically, participants were identified as cases or controls based on scores from the Word Attack non-word reading (cases: $\overset{-}{x}=86.57,\pm 10.32$; controls: $\overset{-}{x}=110.33,\pm 11.04,$ t=−15.69, p<2e−16), Word Identification real-word reading (cases: $\overset{-}{x}=81.66,\pm 11.04$; controls: $\overset{-}{x}=111.94,\pm 12.19,\;t=-17.86,$ p=<2e−16), and Passage Comprehension (cases: $\overset{-}{x}=84.93,\;\pm 13.18$; controls: $\overset{-}{x}=108.85,\pm 9.76;$; t=−3.527,, p=0.0005) sub-test measures from the Woodcock-Johnson IIIR or Woodcock Reading Mastery Tests (Woodcock, 1987; Woodcock et al., 2001), Verbal Comprehension measure (cases: $\overset{-}{x}=102.09,\pm 13.09$; controls: $\overset{-}{x}=118.15\pm \;13.05;\;t=-8.507,$ p=5.26e−15) from the Wechsler Intelligence Scales for Children or the Wechsler Abbreviated Scales of Intelligence (Wechsler, 1999; 2004), and rapid naming (cases: $\overset{-}{x}=95.26,\pm 23.66$; controls: $\overset{-}{x}=102.88,\pm 11.63,\;t=-2.834,\;p=0.005$) from the Comprehensive Test of Phonological Processing Rapid Automatized Naming or the Rapid Alternating Stimulus Tests (Wagner et al., 1999; Wolf and Denckla, 2005).

The distributions of scores from the behavioral tests across participants are shown in Results and demonstrates a typical heterogeneous pattern of reading disability, with a relatively greater frequency of children with poor phonological decoding and fewer cases with poor reading whose performance was low across the measures and cases with a relatively specific problems with reading comprehension (Eckert et al., 2017). The behavioral profile of the reading disability group is consistent with the “specific learning disorder” and the “developmental learning disorder for reading” diagnoses from the DSM5 and the World Health Organization (ICD-11), respectively. Both of these definitional frame-works include a criterion for persistent impairment. We use the term reading disability for short-hand, to differentiate from a dyslexia term that has often been operationally defined in imaging studies and could include a reading skill and cognition discrepancy, and because we did not have data about the duration of reading skill impairment in the sample.

### 2.3. Demographically balanced reading disability and control groups

Each case was selected to be matched with a sex-matched control with a similar age from cases in the multi-site retrospective dataset. That is, there were an equivalent number of males and females in each group and the groups did not significantly differ in age (*F*_1, 190_ = 0.39, *p* = 0.535). This selection process resulted in group differences in the frequency of cases and controls from each contributing research site (*χ*^2^_7_ = 18.11, *p* = 0.012). For this reason, research site was considered in the approach used to train the reading disability classifiers and in post-hoc analyses to evaluate reasons for varied classification probability across participants.

### 2.4. Image acquisition and processing

Supplementary Table 1 presents image acquisition information for the T1-weighted images that were used in this study. These images were denoised (Manjon et al., 2010), bias field corrected using the Statistical Parametric Mapping (SPM12) non-uniformity correction function, and rigidly aligned to the MNI 152 T1 1 mm template using the SPM co-registration function. These images were then segmented using the SPM12 segmentation function to generate gray matter, white matter, and CSF images. The summed total gray and white matter volumes were larger in the control group compared to the reading disability group (*F*_1, 190_ = 5.74, *p* = 0.018). For this reason, the total brain volume measure was also considered in post-hoc analyses to evaluate reasons for varied classification probability across participants.

Jacobian determinant measures of regional volumetric differences across participants were collected from the T1-weighted images. These Jacobian images produce results that are similar to cortical surface area approaches (Eckert et al., 2019), and allowed for the examination of voxels across gray and white matter regions. The Jacobian determinant images were obtained with the following procedures. The segmented gray and white matter images were normalized to a sample-specific coordinate space using SPM12 Diffeomorphic Anatomical Registration Through Exponentiated Lie Algebra (DARTEL) normalization (Ashburner et al., 2007). A sample-specific template space was used for this pediatric study to optimize the normalization of images to a common coordinate space (Wilke et al., 2003). The default SPM12 settings were used across image processing steps because the settings were designed to work with T1-weighted images from multiple different research sites and to facilitate replication (Kurth et al., 2015). Jacobian determinant images were generated from the normalization parameters. The Jacobian determinate quantifies the extent of volumetric expansion and contraction needed to warp an image to the sample-specific template. That is, we sought to optimally normalize the images to a common coordinate space to measure how much volumetric displacement across gray matter and white matter regions was necessary to align the images. Finally, the Jacobian images were smoothed with an 8 mm full-width-half-maximum Gaussian kernel, log-transformed to remove skewness across voxel values, scaled between 0 and 1 to normalize the data across our entire dataset, and then masked with *a* > 0 threshold to exclude the contribution of non-brain regions (empty space) in the analyses. These 121 × 145 × 121 images, representing regional brain structure volume differences, were used in the deep learning methods described next.

### 2.5. Hybrid unsupervised and supervised classification approach

**Fig. 1.**
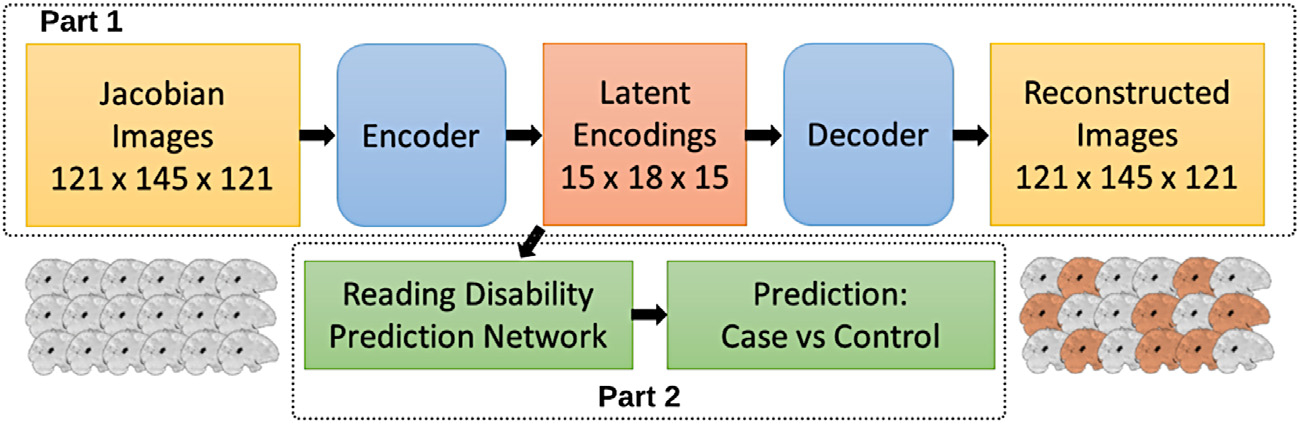
Overview of the AE (Part 1) and CNN (Part 2) approach. We first generated latent representations of the Jacobian determinant images of voxel-wise expansion and contraction required to normalize each image to a study-specific template. These latent representations were used as training input to a CNN model to predict reading disability cases from controls.

Fig. 1 provides an architectural overview of the AE data reduction and CNN classification approach used in this study. We leveraged the advantages of unsupervised and supervised deep learning methods to deal with the challenges of high dimensionality and phenotypic heterogeneity facing classification studies of dyslexia neuroimaging data using a two-step approach. That is, we 1) trained a stacked AE to identify latent representations of the brain images and then 2) used the latent representations as input to train and test a CNN model to classify individuals as reading disability cases or controls. These latent representations represent key features in the images that characterize different patterns of variance. This approach is similar to a principal components analysis where the dimensionality of many colinear variables can be reduced to a smaller set of key components or features that represent much of the variance across variables.

#### 2.5.1. Latent representation using autoencoders

AEs have been in use for many years (Ballard, 1987; Rumelhart et al., 1985) and are widely used in machine learning research (Gosztolya et al., 2019; Ha and Schmidhuber, 2018; Kusner et al., 2017). They have been mainly used in Computer Vision and other fields because of their simple implementation relative to their strengths in reducing dimensionality (Newell and Deng, 2020), feature learning, generative modeling, etc. (Suk et al., 2015). The previous success of AEs across classification domains provided the rationale for their use in data compression and extracting low dimensional features from brain images, which were subsequently classified in the current study.

The internal architecture of the AE network is divided into encoder and decoder functions. The encoder function transforms image information X into a latent representation, Z=Encoder⁡(X). The decoder function reconstructs or approximates the original image $X^{'}$ based on the latent representation, $X^{'}=Decoder(Z)$. For our purposes, the encoder function compressed each Jacobian determinant image X (of shape 121 × 145 × 121) into a lower dimensional representation Z that consisted of 32 volumetric feature maps of shape 15 × 18 × 15. The decoder function was parameterized by weights that project the low dimensional representation Z back to the original dimensions of the input image $X^{'}$. The parameters in the encoder and decoder network were optimized jointly to minimize the reconstruction loss J from the objective function, $J=\Sigma {\left|X-X^{'}\right|}^{2}$, over all training examples. This algorithm produces weights for the encoder and decoder functions that are optimized to minimize reconstruction losses.

We used a 3-layer parameterized 3D Convolutional Neural Network as the encoder function that applies spatially sensitive convolution-driven non-linear transformations to the original image to construct the low dimensional latent representation. For the decoder, we used a 3-layer 3D Deconvolutional Neural Network (Zeiler et al., 2010) to reconstruct the original image back from the compressed latent space. Whereas the encoder down-samples and compresses the data to a lower dimensional representation, the decoder up-samples the data to the original dimension. That is, the compressed latent representations from the encoder model were used to reconstruct the original image. Concretely, the size of the latent representation (Z) was significantly smaller (compressed size of 7.325%) than the original brain image (X), which in technical terms is an “under-complete” AE (Goodfellow et al., 2016). The AE used here effectively aggregates the information from various local regions of the brain and represents these regions in a low dimension by finding local correlations in the brain images that could be represented as principal components. Finally, the latent representations from the AE unsupervised learning step were used as input for supervised training. Here we chose a 3-layer 3D CNN architecture for the supervised learning task. The network outputs a single logit as a non-linear function of the latent encoding.

### 2.6. Training and implementation

#### 2.6.1. Training the autoencoder

**Fig. 2.**
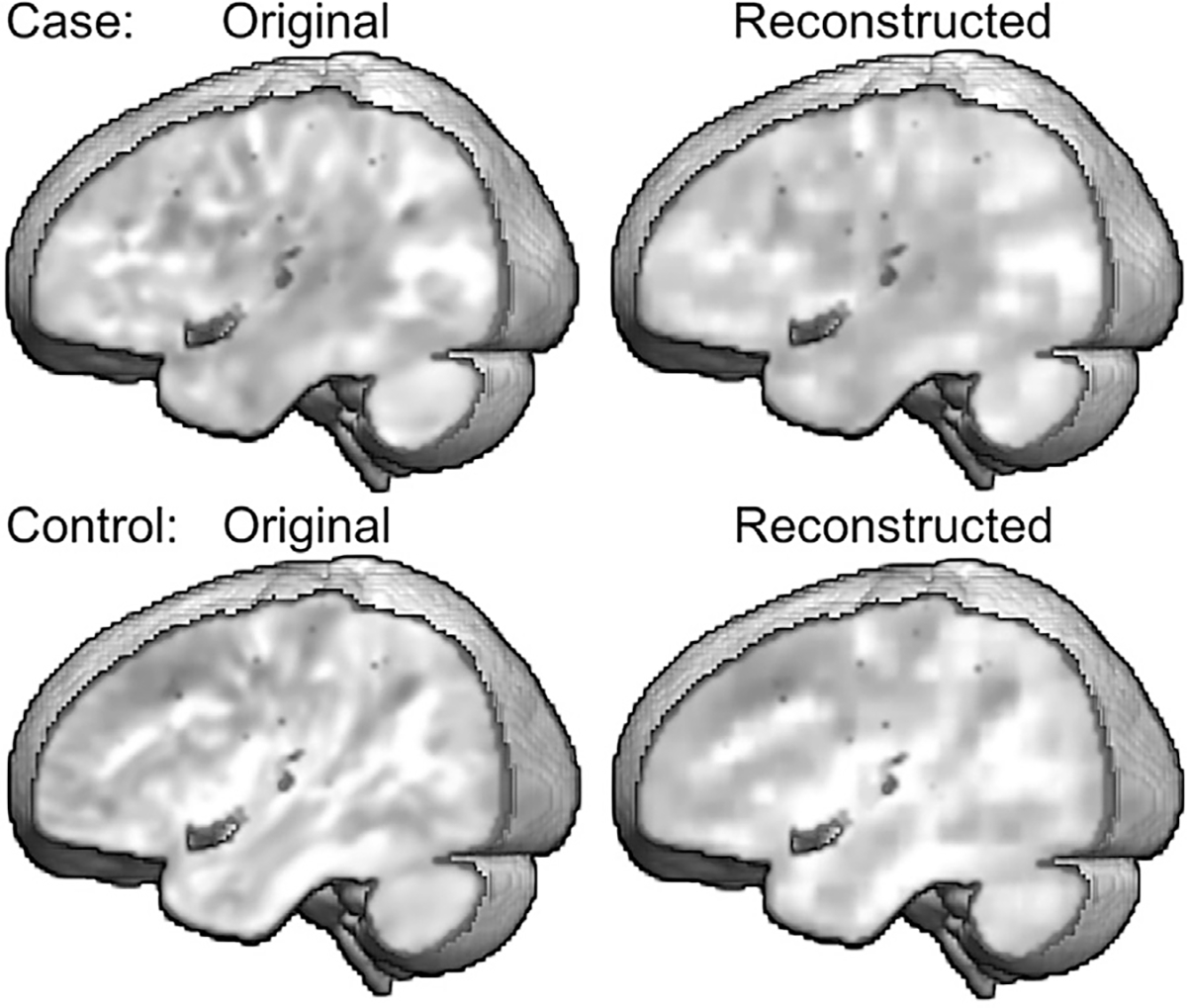
Accurate reconstruction of the original Jacobian determinant images was observed across participants and shown here for a reading disability case and control. Bright regions had to be reduced in volume to fit to the template. Smoothing of the reconstructed images was the primary difference with the original images.

AE training included randomly splitting the dataset into train and test samples of 153 and 39 images, respectively. Again, the AEs included three layers to encode and decode the images. Rectified linear unit (ReLU) activation (Nair and Hinton, 2010) was used to represent the data for the first two levels of the decoder, and sigmoid activation was applied to the final layer. In our experiments, we found that a reduced feature map containing 32 channels of size of 15 × 18 × 15 (approx. 6% of the 121 × 145 × 121 image) provided the optimal reduction in dimensionality that was sufficient for the decoder to reconstruct the original image. Each of the 32 channels captures spatial features of the input image in lower-dimensional volumetric feature maps (of size 15 × 18 × 15) that can be used to reconstruct the original image with minimal loss of information by the decoder. Reconstruction error, calculated as the mean square error between the decoder output and the original brain image, converged to a loss of 10^−5^ after 2500 training steps, which showed that the original input was represented well by the latent representations or components generated by the encoder. Fig. 2 shows the high correspondence in the distribution of voxel values across original and reconstructed images for a reading disability case and control.

#### 2.6.2. Training the reading disability prediction neural network

To increase the likelihood for stable classification accuracy, independent of the research site, we employed a stratified train-test split ratio where the numbers of cases and controls from each research site were approximately equal across sites (Mohajer et al., 2020). The model was completely site-agnostic, and no site-dependent processing was required for the brain images. The CNN model consisted of one 3D convolution layer (Python3 – Tensorflow version 1.15, Conv3D) (Abadi et al., 2016) which took input as the latent encoding of shape 15 × 18 × 15 with 32 filters and outputs feature maps of size 7 × 8 × 7 with 24 filters. These images were then flattened and passed through a fully connected layer of 128 neurons with ReLU activation. The final layer contained one neuron optimized to predict binary dyslexia and control class labels. The model weights were trained using the Adam Optimizer (Kinga and Adam, 2015) with a learning rate of 0.001. To prevent overfitting, the test-set loss was tracked after every 200 model update steps and training was stopped early when the test-set loss diverged from train-set loss. Batch normalization was used on each layer of our network, which boosted training speed and made the training less sensitive to parameter initialization. Dropout layers (Srivastava et al., 2014) were also used after each batch normalization layer to limit over-fitting, which involved randomly ignoring a subset of voxels in intermediate feature maps during training. Model generalization with the test data was optimal when the dropout rate, a hyperparameter that determines the probability of ignoring a voxel during a training step, was set between 0.5 and 0.6. Note that no dropout was used during prediction on the test set.

#### 2.6.3. Comparison to alternative classification and data reduction approaches

Analyses were also performed to compare the deep learning results to other standard dimensionality reduction methods, such as Principal Component Analysis (PCA) and supervised machine learning approaches, such as SVM and RF that have been used in previous studies using neuroimaging data to classify images from dyslexia studies (Cui et al., 2016; Płoński et al., 2017; Tamboer et al., 2016; Usman et al., 2021). To compare the latent features extracted by our AE with the PCA features, we trained traditional supervised machine learning algorithms like SVM and RF to train a binary classifier for predicting reading disability. To perform PCA, we first converted the 3-D brain volume image into a 1-D vector array and then used Singular Value Decomposition (SVD) to reduce the dimensionality such that 90% of the variance of the original data was still retained. The reduced data was then used to train the supervised classifiers mentioned above. The AE training method outputs a 4-D latent space (32 feature volumes each of size 15 × 18 × 15) and traditional SVM and RF methods expect the input data to be 1-dimensional. Hence, we flattened the AE-learned latent space into a 1-D array to train the supervised machine learning models. Additionally, to understand the impact of dimensionality reduction on the 3D CNN results, we trained another 3-D CNN to directly learn from the original full-dimensional data without doing any dimensionality reduction. We used the default parameters for SVM (https://scikit-learn.org/stable/modules/generated/sklearn.svm.SVC.html) and RF (https://scikit-learn.org/stable/modules/generated/sklearn.ensemble.RandomForestClassifier.html) as listed in the scikit-learn documentation (Pedregosa et al., 2011). The SVM and RF supervised methods were compared across PCA and AE approaches, relative to the CNN approach with and without the AE approach.

#### 2.6.4. Importance features: Brain regions underlying classification

The most informative voxels for accurate classification of reading disability cases and controls were identified based on an established image perturbation method (Ribeiro et al., 2016) that leveraged the trained AE and reading disability classification networks. First, we randomly generated 10,000 noise filters $\left(K_{j}\right)$ of the same dimension (121 × 145 × 121) as the Jacobian images. For each noise filter, we randomly selected a contiguous region (s) of 8 × 8 × 8 and subsampled those values weighted by a mean-centered unit Gaussian distribution $\left(K_{js}\right)$. The process was iterated across the space of each Jacobian image $\left(X_{i}\right)$ in the dataset, applying each subsampled noise filter to the image as follows: $F_{ijs}=X_{i}+K_{js}$, where $X_{i}$ is the $i^{\text{th}}$ subject from the dataset and $K_{js}$ is the $s^{\text{th}}$ subsample of the $j^{\text{th}}$ noise map. We then computed the dyslexia classification probability $y_{ij}$ after each perturbed image $F_{ijs}$ was input to the encoder function from the pre-trained AE and classified the resultant latent image $Z_{ijs}$ with the pre-trained CNN model.

Classification error $\left(e_{ijs}\right)$ was calculated as the difference in reading disability case classification probability $\left(y_{ijs}\right)$ for the perturbed image $\left(F_{ijs}\right)$ compared to the base probability $\left(y_{i}^{*}\right)$ for the original, unperturbed image $\left(X_{i}\right)$, such that $e_{ijs}=y_{i}^{*}-y_{ijs}$. Since each noise filter perturbed a specific 8 × 8 × 8 region and the rest of the image was unchanged, classification error $y_{ijs}$ represents how much of an impact the space filtered by $K_{js}$ had on the reading disability classification accuracy for image $X_{i}$. For example, if an image $X_{i}$ was from a reading disability case $\left(y_{i}^{*}=1\right)$ and the noise added $F_{ijs}$ had a lower classification probability $(y_{ijs}\langle 1;\;e_{ijs}\rangle 0)$ then the features/voxels from the perturbed regions are inferred to decrease sensitivity to reading disability. Likewise, if an image $X_{i}$ was from a control subject $\left(y_{i}{\;}^{*}=0\right)$ and the classification probability increased $\left(y_{ijs}>0\right)$, then the error would be negative $\left(e_{ijs}<0\right)$, which would suggest that the perturbed region $\left(K_{js}\right)$ limited sensitivity for controls. Finally, error values close to zero would indicate that classification was not strongly affected by a perturbed region, since the classification probability was changed minimally by noise perturbations.

A sensitivity map $S_{i}$ was generated for each brain image $X_{i}$ by accumulating the voxel-specific error score $\left(e_{ijs}\right)$ in the space of each filter $\left(F_{ijs}\right)$. Concretely, the sensitivity map $S_{i}$ is of the same dimension as the original brain image (121 × 145 × 121), which summarized how each voxel affected the classification score of the original image. The $S_{i}$ image was initialized as zeros. At each iteration of applying $K_{js}$ and obtaining $e_{ijs}$, we incremented all the values of $S_{i}$ located in the perturbed voxels of $K_{js}$ by $e_{ijs}$. We normalized *S*_*i*_ by dividing each voxel-value by the number of times that the corresponding voxel was selected for perturbation. Since increased absolute model prediction error indicated that the perturbed regions influenced classification accuracy, the sensitivity map summarizes the importance of each voxel for predicting the corresponding Jacobian image. That is, the perturbation approach quantified the importance of each voxel for the classification of a participant’s image.

The above process was repeated for each of 192 brain images to obtain importance or sensitivity estimates for all voxels within each participant, again based on the amount of classification error observed for that participant. These importance maps were then averaged at each voxel across reading disability cases and across controls to identify brain regions that were consistently more important for classifying reading disability cases and for classifying controls. As shown later in Results, different brain regions had different importance in accurately classifying cases and classifying controls. A 25% change in classification accuracy was used to identify specific brain regions where the average Jacobian determinant value was collected to further investigate the contribution of these brain regions to classification accuracy.

#### 2.6.5. Machine learning implementation

We used the Python3 programming language for data analysis and visualization. The CNNs were implemented using Tensorflow 1.15 (Abadi et al., 2016). The PCA, SVM, and RF approaches were implemented using the Scikit Learn Machine Learning Library (Pedregosa et al., 2011). Training of the AE model took ~8–12 h using an Intel Xeon processor with 24 cores running at 2 GHz and 32 GB of RAM and 1 V100 GPU with 2880 CUDA cores and 12 GB of RAM, which were available from the Clemson University Palmetto cluster. A dedicated GPU was used to accelerate the training process.

### 2.7. Statistical analyses

Receiver operator characteristic (ROC) analysis was performed to evaluate CNN test classification accuracy. Here, 100 random train-test cross-validation splits (stratified by site) were created with a test size ratio of 20%. For each split, a CNN classifier model was examined with randomly initialized weights. Only the CNN of the AE + CNN approach was trained for each split. Overall accuracy (percent correct across the sample), precision (true positives / (true positives + false positives)), and recall (true positives / (true positives + false negatives)) metrics were collected to evaluate the classification results. Precision is a metric of accuracy that accounts for the false positives or when an image from a control was labeled as a reading disability case. Recall is a metric of accuracy that accounts for false negatives or when an image from a reading disability image was labeled as a control.

Conventional general linear model analyses were also performed following the deep learning analyses with an emphasis on evaluating the classification results. First, *t*-test comparisons were performed to examine the impact of machine learning method on classification accuracy. Regression analyses were then performed to examine the associations between reading skill measures and the AE + CNN classification probability across participants. Here the median classification probability was examined for each participant, which was obtained by randomly selecting each participant for test classification (5 – 30 times, smaller sample ratios for research sites with larger sample sizes, as part of the approach to controlling the site effects in the development of the classifier). Regression and Pearson correlation were also performed to examine the relative contributions of specific brain regions, those identified with the image perturbation approach for feature importance, to the classification probability variable. Finally, a regression control analysis was performed to determine how demographics, research sites, and total brain volume influenced the classification results.

## 3. Results

### 3.1. CNN test classification accuracy and comparison with other methods

**Fig. 3.**
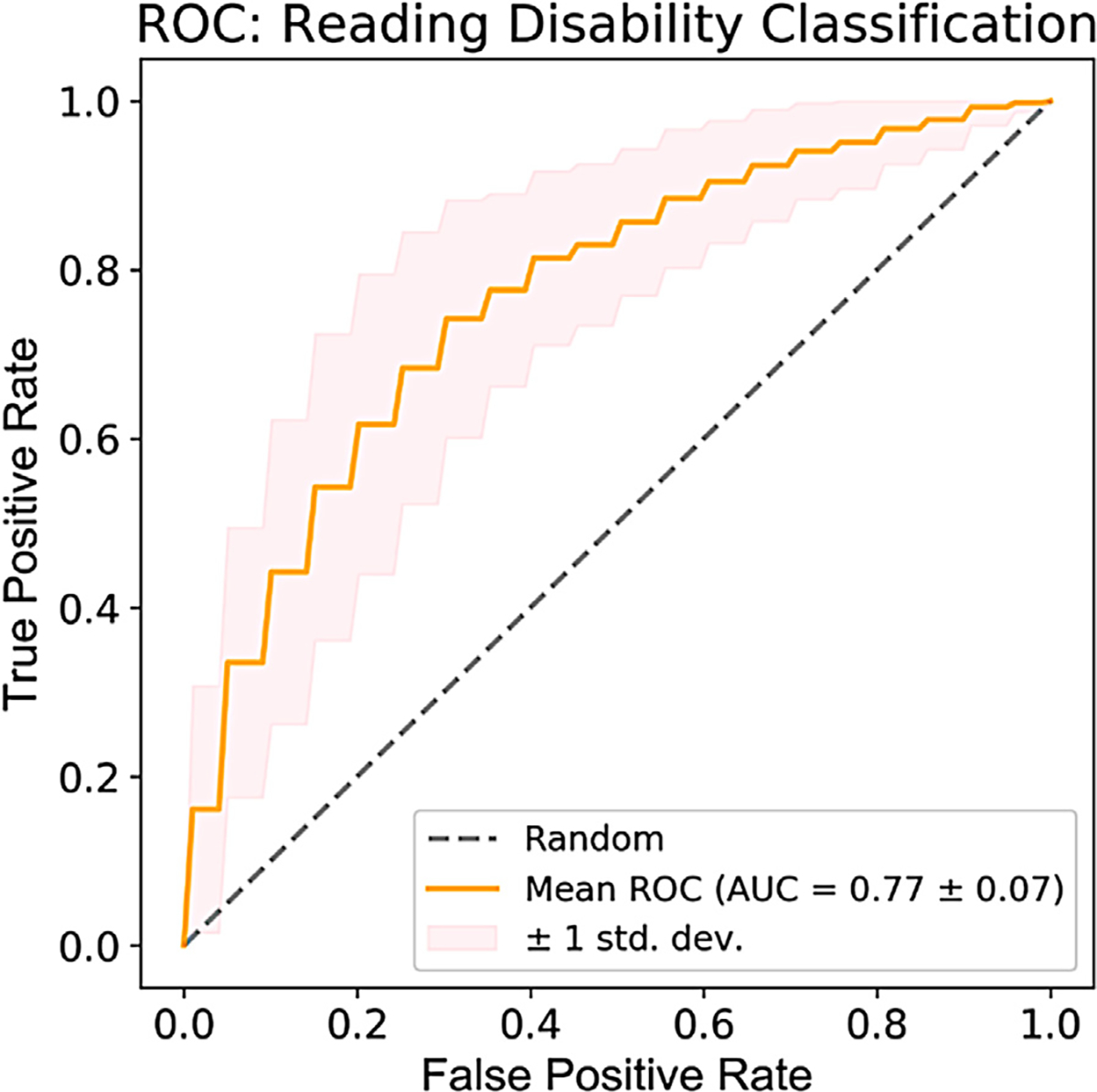
Receiver operating characteristic (ROC) plot showing the mean (± 1 std dev) false positive and true positive classification rates across 100 random train-test splits.

The mean area under curve (AUC) for the AE + CNN models was 0.77 ± 0.07, as shown in Fig. 3. The combined AE + CNN approach demonstrated up to 85% correct classification accuracy on individual classification runs. The precision of reading disability classification was 0.75 ± 0.08 and recall of a reading disability classification was 0.78 ± 0.12.

**Fig. 4.**
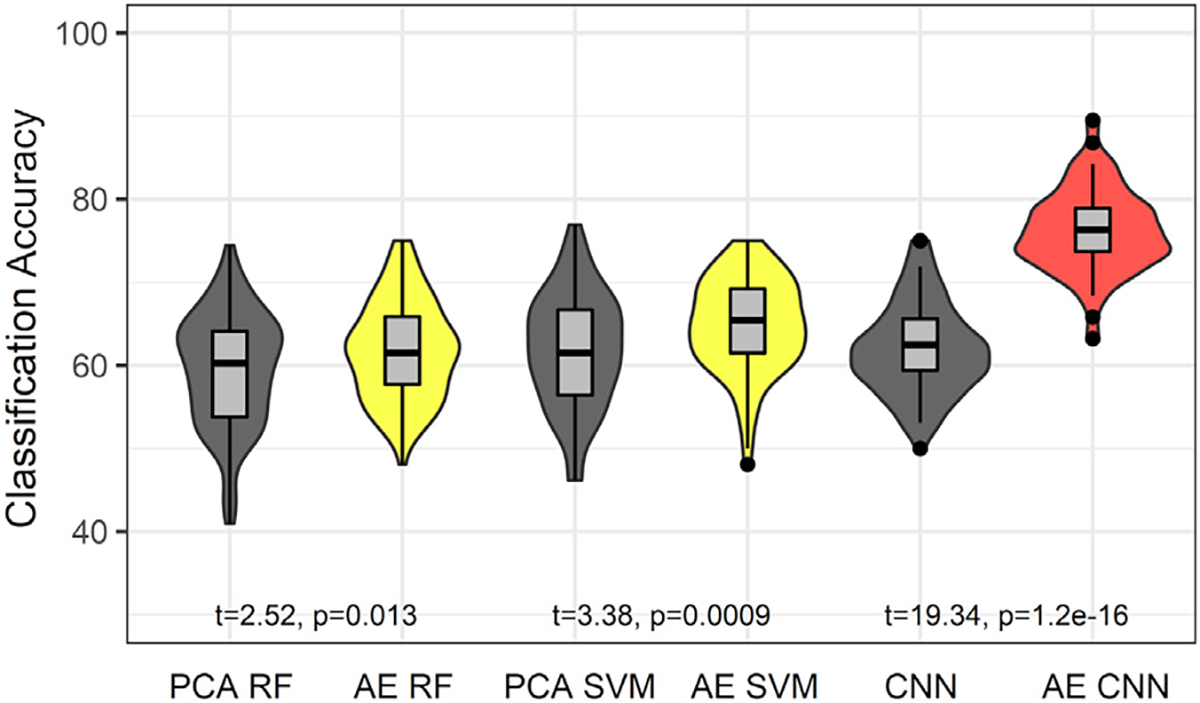
Test sample classification accuracies across data reduction and machine learning methods demonstrate the classification advantage of using a combined AE + CNN method compared to RF and SVM. Paired *t*-test statistics shows significantly higher classification accuracy for each machine learning method when AE was used compared to PCA. In addition, large effect sizes were observed for comparisons of the AE + CNN (red) method with every other method (Cohen’s *d* = 2.16 to 2.93).

The AE + CNN approach demonstrated superior performance compared to the SVM and RF classification methods. For example, the SVM classifier achieved a mean test accuracy of ~61%, while the Random Forest classifier achieved mean test accuracy of ~59%. These results were due in part to benefit from the AE. Paired *t*-test of the classification accuracies between methods demonstrated significantly higher accuracy across machine learning methods when the AE was used in comparison to PCA for reducing the dimensionality of the data and when no data reduction was used for the CNN analysis, as shown in Fig. 4.

### 3.2. Individual differences in reading skills and classification probability

**Fig. 5.**
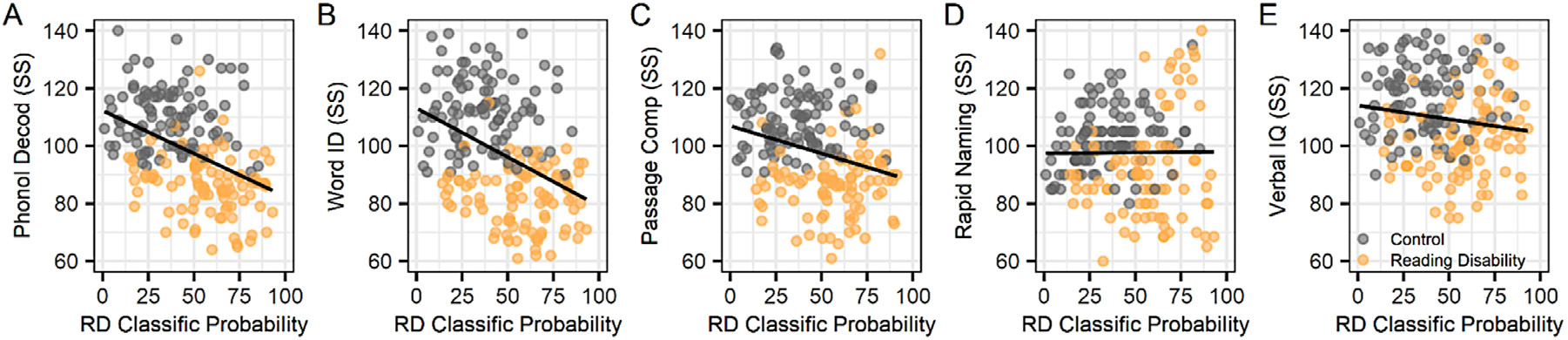
Brain morphology classification depended on differences in non-word (phonological decoding) and real word reading. The x-axis (RD Classific Probability) indicates the median classification accuracy for each participant across their repeated random sampling for model testing. Reading disability cases (orange) and controls (gray); standard scores (SS).

Fig. 5 shows the individual reading-related scores for each participant to demonstrate the varied abilities in this sample. It also shows that the probability of a reading disability classification increased with lower non-word reading accuracy (*r*_190_ = −0.39, *p* = 8.872e-08), real word reading accuracy (*r*_190_ = −0.38, *p* = 2.877e-07), and to a lesser extent for passage comprehension (*r*_190_ = −0.33, *p* = 1.051e-05), rapid naming (*r*_190_ = −0.08, *p* = 0.313), and verbal comprehension (*r*_190_ = −0.23, *p* = 0.003). That is, children on the opposite ends of the non-word and real word reading distribution were most clearly differentiated using the AE + CNN classifier.

### 3.3. Brain regions contributing to classification accuracy

**Fig. 6.**
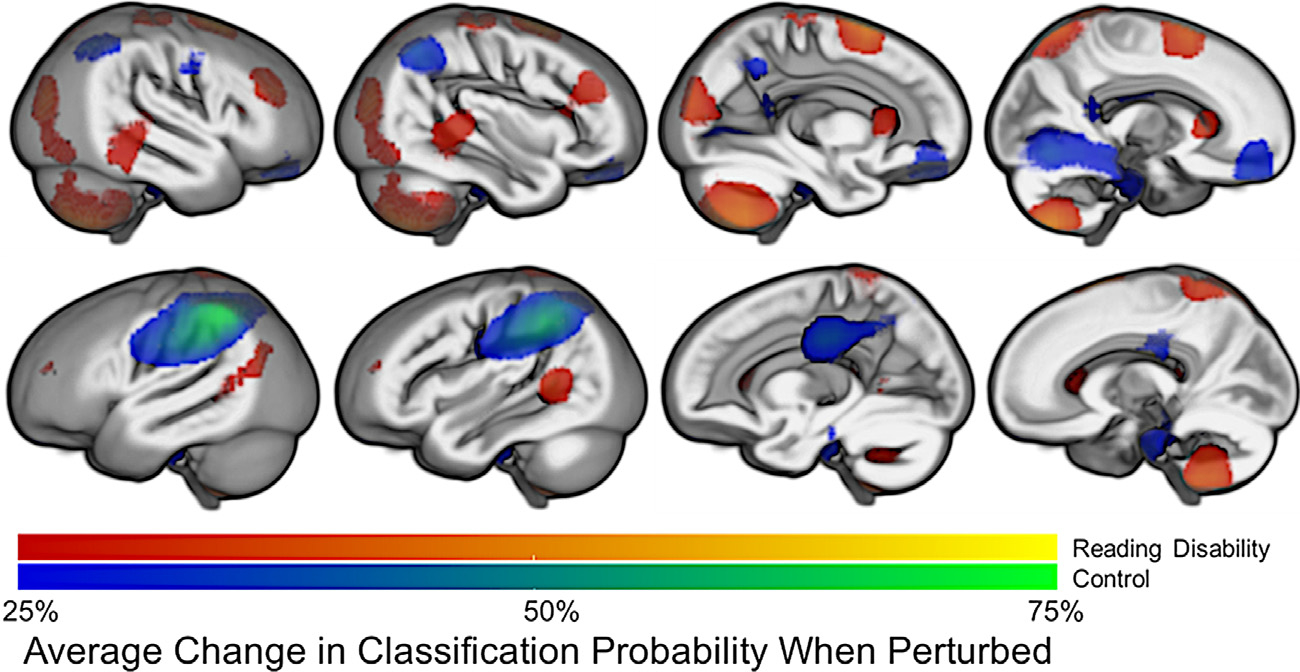
Brain regions that were important for AE + CNN classification. The probability of reading disability (red-yellow) or controls (blue-green) decreased when spatially-specific voxel values were perturbed. For example, a 75% decrease in reading disability or control classification accuracy corresponds to a change in probability from 100% to 25% and this would be represented by yellow or green, respectively

The image perturbation approach allowed for the differentiation of regions where a subset of reading disability cases or controls exhibited Jacobian values that were particularly different from the comparison group, which would explain why one brain region was more sensitive for identifying reading disability cases or identifying controls. This approach demonstrated that widespread brain regions contributed to classification accuracy. Fig. 6 shows that reading disability classification accuracy depended on superior temporal sulcus, cerebellar hemisphere, superior frontal, and lateral visual cortex regions, whereas control classification depended on the left inferior parietal lobule, orbitofrontal, and lingual gyrus regions.

To further investigate the contribution of specific brain region(s) to classification accuracy, multiple regression was performed using the average Jacobian determinant values from the regions shown in Fig. 6 (e.g., right caudate, left inferior parietal lobule, right orbitofrontal, right lingual gyrus, and left superior temporal sulcus). Across the entire sample, these region of interest variables explained 42% of the variance in classification accuracy (*F*_14, 177_ = 9.19, *p* = 5.258e-15), with the left inferior parietal lobule (*Beta* = −1.11, *t* = −3.74, *p* = 0.0003), right orbitofrontal cortex (*Beta* = −0.50, *t* = −2.53, *p* = 0.012), and left superior temporal sulcus (*Beta* = 0.46, *t* = 2.48, *p* = 0.014) explaining significant unique variance. While this is an imperfect linear representation of the non-linear AE + CNN results, the regression results suggest at least some dependency on these brain regions for classification. Table 1 presents Pearson correlations for the classification probability, these uniquely predictive brain regions, and the behavioral measures.

### 3.4. Limited impact of demographic and research site factors

Model training was performed using site stratification, thereby limiting the influence of research site effects on the classification models. Site effects were observed in the model testing results where classification probability appeared to depend on research site (F_7, 184_ = 9.23, *p* = 8.906e-10), but this result was due in large part to differences in the composition of the test data rather than the training data. More specifically, the site association with test classification probability appeared to be due largely to one research site that had more reading disability cases (*N* = 22) than controls (*N* = 4), as well as significantly lower non-word reading scores compared to the other sites (*t* = −6.304, *p* = 3.66e-09), in contrast to reading comprehension scores (*t* = 3.733, *p* = 0.520). That is, the classifier appeared to be an effective predictor of reading disability rather than confounded by site because of the stratification approach used to train the AE + CNN model.

Pearson correlation also demonstrated that older children (*r*_190_ = 0.18 *p* = 0.013) and those with lower total brain volume (*r*_190_ = −0.44, *p* = 1.756e-10) had higher test classification probabilities. Sex was not significantly related to classification probability (*r*_190_ = −0.04, *p* = 0.560). To further investigate the extent to which these variables and research site accounted for the probability of classification, they were included in a regression analysis with the test classification probability for reading disability or control status. Classification probability was an expected significant predictor of group status (*t* = 4.99, *p* = 1.39e-06), but sex/gender, age, research site, and total brain volume were not significant predictors of group status (*p*s > 0.216). These results show that while age, total brain volume, and research site were related to classification accuracy, the locally specific brain regions identified as important for AE + CNN classification were independently critical for classification accuracy.

## 4. Discussion

The results of this study demonstrate that a combined AE + CNN deep learning approach optimized classification accuracy based on neuroimaging data, and that perturbing the voxel values can highlight brain regions critical for identifying disability/disorder cases or for identifying controls. Specifically in the context of reading disability, the AE + CNN classifier included features or brain regions of interest that have been reported as structurally atypical in previous studies, such as left superior temporal sulcus and lingual gyrus effects described in meta-analyses of the voxel-based gray matter literature (Eckert et al., 2016; Richlan et al., 2013). Findings from the current study provide an analytical framework for the development of classifiers in large samples that may be useful in identifying atypical brain regions in children with different reading skill deficits.

### 4.1. Classifier optimization

Fig. 4 clearly shows that the AE approach enhanced classification accuracy across the SVM, RF, and CNN approaches. Given a relatively modest sample size, the AE appeared to help deal with the “curse of dimensionality”. Only 32 filters were necessary to accurately represent the Jacobian images in comparison to the analysis of thousands of voxels or hundreds of brain regions of interest that typically exceed a sample size. That is, the AE helped deal with the well-established problem that training a model is ineffective when there are too few cases to identify the critical and perhaps interacting features among many variables. This was a problem even for the CNN approach when the classification was performed with no AE. CNN classification accuracy was not meaningfully better than the SVM and RF classification accuracies when the AE was not included in CNN classification. Other types of AEs, like denoising or variational AEs, may provide further benefit and should be considered in future studies

The CNN approach meaningfully outperformed the other classification approaches when combined with the AE. Here the capability of the AE to generate latent representations of complex spatial variation in brain structures allowed the CNN to encode and use this spatial variation to learn the group labels more effectively than the other approaches. Neural network architectures are traditionally known to be “data-hungry”, often requiring millions of examples to learn robust and generalizable representations. Here, we demonstrated a simple two-step data-driven architecture with unsupervised feature extraction combined with supervised deep learning to provide optimal results on, by deep learning standards, a small dataset of 192 images. Further optimization may also be possible with both the AE and the classifier components of the architecture, particularly with more training data being available. A denoising autoencoder (Vincent et al., 2010), where the autoencoder training requires the model to reconstruct the original image from the perturbed image has been shown to be a robust alternative to the traditional autoencoders used in this paper. Residual connections, which have proven to be successful in image recognition tasks (He et al., 2016), could be used between CNN layers to train even deeper neural networks. Instead of optimizing for reconstruction loss, training with an auxiliary contrastive loss function (Hadsell et al., 2006) that is designed to cluster cases and control images in the latent space can also be a future avenue for improvement. In addition, an ensemble of multiple CNNs on different image features that includes different imaging modalities or structural measures may be able to improve classification accuracy and robustness.

### 4.2. Research site effects

There is increasing availability of large multi-site datasets (e.g., ADNI, Human Connectome, ABCD Studies) where data were collected with common protocols but may still vary due to factors that cannot be controlled by researchers, such as site differences in participant enrollment or the lifecycle of scanner hardware. Similarly, multi-site retrospective data repositories will have inconsistent protocols for sampling or imaging across sites (e.g., ABIDE, ENIGMA). Our multi-site retrospective dataset included sites with different numbers of subjects. Initial analyses that did not consider the effect of research sites led to site-biased results that did not generalize across sites (results not shown). While there appeared to be a site effect on the test classification results, this effect was driven by one site with a small number of subjects and over-representation of reading disability cases compared to controls. That is, rather than a site effect, the classifier was an effect predictor of the reading disability cases from this site. The use of stratification by site may be useful in dealing with multi-site effects in the analysis of other open access datasets.

### 4.3. Multi-dimensional classifier

Here, we used a group classification approach across machine learning methods to differentiate cases from controls. However, the results suggest that there may be additive value in creating a machine learning predictor of phenotypic dimensions of a disorder. This premise is supported by the region of interest results from this study where specific brain regions exhibited relatively specific associations with reading-related skills. For example, lower right caudate Jacobian values were associated with lower passage comprehension. A similar association was observed in children with low right caudate activity during a reading comprehension task and differences in a reading comprehension standardized assessment (Aboud et al., 2016). The results of the current study appeared to include multiple brain regions because of differences in how those regions relate to different reading-related abilities (Table 1).

One reason for the multidimensional influence on the classifier results is of course the inclusion of control participants who had relatively higher reading-related scores compared to the reading disability cases, as shown by the large effects in Table 1. Thus, interpretation of the classifier results should also be considered in the context of the controls who exhibited varied reading-related skills within the average to the above-average range. This perspective is supported by the relatively high classification accuracy for the controls (79% accuracy). That is, the classifier was effective at identifying children with above-average reading-related scores and not just the children with below-average reading-related scores. These results are consistent with a previous dyslexia classification study showing that classification accuracy based on white matter measures was significantly related to performance on a word list reading task (Cui et al., 2016).

### 4.4. Image perturbation and individual cases

We were able to identify specific brain regions that were important for the classification of each participant based on an image perturbation approach (Ribeiro et al., 2016). Perturbing voxel values as part of a classification pipeline has the potential for clinical application because this approach can be used to identify the brain regions that were critical for classifying each participant. This type of information could be coupled with behavioral data for a differential diagnosis. For example, two children may have the same phonological processing deficits but exhibit spatial differences in the location of atypical brain structures (e.g., the auditory versus visual cortical regions in Fig. 6). A deep clustering approach, where the latent representations from the autoencoder are used to differentiate cases with unique anatomical and behavioral profiles, may be useful in future studies. This approach contrasts with the group-level neuroimaging approaches that are typically used to make statistical inferences about population-level differences. The classification approach shown here is a cautious first step towards the goal of differential behavioral and anatomical diagnoses that may enhance understanding of an individual’s reading disability and help guide intervention planning and evaluation of outcomes.

### 4.5. Relevance to the dyslexia structural imaging literature

In the context of voxel-based gray matter studies, the current results are consistent with atypical morphology findings in the superior temporal sulcus, inferior parietal lobule, orbitofrontal gyrus, lingual gyrus and cerebellum (Eckert et al., 2016; Eden et al., 2016; Linkersdörfer et al., 2012; Richlan et al., 2013; Vandermosten et al., 2016). The superior temporal sulcus specifically, appeared to be relatively specific to the reading disability sample based on the image perturbation results. That is, atypical morphology in this region may contribute to or be a consequence of reading disability. Atypical posterior superior temporal sulcus structure and function are expected in people with reading disability given that this region is sensitive to phonological features of speech sounds (Vaden et al., 2010) and contribution to categorizing of speech sounds (Liebenthal et al., 2010). Thus, it is not surprising that activity in the superior temporal sulcus has also been reported to be atypical across studies [(Richlan et al., 2009; Maisog et al., 2008; Salmelin and Helenius, 2004); although perhaps only in studies of adults (Richlan et al., 2011)]. Moreover, these structural and functional effects appear to occur across morpho-syllabic languages than in alphabetic languages (Yan et al., 2021).

There were some findings that were unexpected, including multiple right hemisphere regions that differentiated reading disability cases from controls. While there have been reports of right hemisphere gray matter findings in reading disability studies (Eckert et al., 2005; Krafnik et al., 2014), these have been inconsistently reported compared to left superior temporal, supramarginal, and orbitofrontal/inferior frontal findings (Eckert et al., 2016). The right orbitofrontal and lingual gyrus regions observed in the current study were important for classification of controls rather than reading disability cases. Thus, some findings in the reading disability literature may depend on characteristics of control samples, as suggested earlier.

The ability to identify regions that were important for classification of control participants has interesting promise for interpreting results and guiding follow-up studies. While speculative, there is the intriguing possibility that above average development of these regions reduces the risk for reading problems. For example, increased goal-directed control through the integration of feedback in orbitofrontal cortex (Rudebeck and Rich, 2018) may support learning to read when effort or cost is required (Plassmann et al., 2007). Or for example, increased working memory capacity (Yue et al., 2019) may allow children to optimally perform grapheme-to phoneme conversion (Costanzo et al., 2012; Sliwinska et al., 2015). It is also possible that the findings reflect proficient reading development, including because inferior parietal lobule volume has been related to literacy/illiteracy (Petersson et al., 2007) and inferior parietal lobule activity during verbal working memory increases from childhood to adolescence (O’Hare et al., 2008) when children are reading to learn. This interpretation is consistent with evidence that middle frontal, cingulate, and orbitofrontal gray matter volume were associated with attention and reading achievement in 233 6–12-year-old children (Wang et al., 2022).

### 4.6. Limitations

As in previous dyslexia classification studies, the size of our sample was relatively modest for a machine learning approach (*N* = 192), and included dyslexia cases with limited diagnostic and reading disability history information. While the cases and controls were matched based on age and sex, and these variables did not appear to substantively affect classification accuracy, a larger sample may be necessary to increase the generalization accuracy. This may include children with varied handedness as we did not characterize potential handedness effects in the current study. Augmentation and synthetic data approaches may be useful for enhancing dyslexia classification methods, including when considering specific reading disability profiles.

Classification accuracy could also be improved with the inclusion of other types of data from T1-weighted images. Here we focused on Jacobian determinant values that characterize the volumetric displacement necessary to align an image to the average brain space of the sample. For example, a relatively large brain region in a participant’s image compared to the rest of the sample would have to be reduced in volume to fit to the template and be represented by relatively large Jacobian values. This approach was useful in previous studies for characterizing group differences in brain structure and we limited the analyses to these data compared to cortical thickness data, for example, to further limit the curse of dimensionality. However, other studies have used cortical thickness regions of interest and white matter metrics (Cui et al., 2016; Płoński et al., 2017), which might be additive with the Jacobian data if latent representations from multiple AEs can be integrated with a deep learning approach.

The integration of functional imaging data may also guide understanding about the theoretical significance of the results. While the extensive functional imaging literature has helped to guide interpretation of the image perturbation results in the previous section, the integration of functional and structural imaging data could provide evidence that atypical morphology in one brain region mediates the relationship between atypical function and atypical reading skills. Moreover, a multimodal approach may help explain why some brain regions that are not considered to be part of a reading network were regions that also contributed to classification.

### 4.7. Conclusions

In a modestly sized multi-site sample of Jacobian determinant (volumetric displacement) images from 192 children, a combined AE + CNN deep learning approach significantly outperformed AE + SVM and AE + RF approaches. While the AE + CNN classification accuracy is relatively high given the sample size and behavioral heterogeneity of the reading disability sample, it is low for a clinically useful tool. Larger samples sizes and quantitative imaging data will be necessary for a stable multi-site classification tool. The strong influence of individual differences in non-word reading and real word reading performance on classification probability suggests that approaches designed to predict differences in reading-related skills may have the potential for clinical application. That is, brain regions contributing to classification may contribute to a differential diagnosis for reading disability for individuals, which considers atypical behavioral tests scores and brain morphology.

## Supplementary Material

1

2

## Figures and Tables

**Table 1:** Means, standard deviations, and correlations with confidence intervals for the reading-related, test classification, and brain regions of interest from the perturbation results shown in Fig. 5.

Variable	*M*	*SD*	1	2	3	4	5	6	7	8	9

1. Group (Control = 0; RD = 1)	0.46	0.50									
	0.45	0.22	.46**								
2. AE + CNN Class. (Control 0 to RD = 1)			[.33, 0.57]								
3. WA	100.12	15.60	−0.73** [−0.79, −0.65]	−0.39** [−0.51, −0.26]							
4. WID	98.60	18.75	−0.79** [−0.84, −0.73]	−0.38** [−0.50, −0.24]	.87** [.83, 0.90]						
5. PC	97.48	16.90	−0.74** [−0.80, −0.66]	−0.33** [−0.45, −0.19]	.72** [.64, 0.78]	.84** [.79, 0.88]					
6. RAN	96.68	16.08	−0.40** [−0.52, −0.27]	−0.13 [−0.28, 0.02]	.30** [.16, 0.43]	.40** [.27, 0.52]	.54** [.43, 0.64]				
7. VIQ	110.13	15.49	−0.57** [−0.66, −0.46]	−0.23** [−0.36, −0.08]	.57** [.46, 0.66]	.56** [.45, 0.65]	.64** [.55, 0.72]	.36** [.22, 0.48]			
8. IPL	1.02	0.12	−0.29** [−0.42, −0.14]	−0.57** [−0.66, −0.46]	.24** [.09, 0.37]	.20** [.05, 0.34]	.26** [.11, 0.39]	.19* [.04, 0.33]	.10 [−0.05, 0.25]		
9. OFC	1.03	0.14	−0.26** [−0.39, −0.12]	−0.56** [−0.66, −0.45]	.23** [.09, 0.37]	.21** [.06, 0.35]	.27** [.12, 0.40]	.16* [.01, 0.30]	.12 [−0.02, 0.27]	.79** [.73, 0.84]	
10. STS	1.01	0.13	−0.13 [−0.27, 0.02]	−0.39** [−0.51, −0.26]	.12 [−0.02, 0.27]	.06 [−0.09, 0.20]	.16* [.02, 0.31]	.17* [.03, 0.31]	.10 [−0.05, 0.24]	.77** [.70, 0.82]	.65** [.55, 0.73]

*Note. M* and *SD* are used to represent mean and standard deviation, respectively. Values in square brackets indicate the 95% confidence interval for each correlation.

* indicates *p* < 0.05.

** indicates *p* < 0.01.

Classif. = Test Classification Probability; IPL = Left Inferior Parietal Lobule; OFC = Right Orbitofrontal Cortex; PC = Passage Comprehension; RAN = Rapid Automatized Naming; RD = Reading Disability; STS = Left Superior Temporal Sulcus; VIQ = Verbal Comprehension WA = Non-word Reading; WID = Real Word Reading.

## Data Availability

The code used in this study is available at: https://github.com/fojoshi/DyslexiaLearn data is available through the Dyslexia Data Consortium: https://www.dyslexiadata.org.
