# Supplementary material for: Deep learning classification of reading disability with regional brain volume features
Source: Neuroimage. Author manuscript; Available in PMC 2023 Jun 1. (PMC10167676; doi:10.1016/j.neuroimage.2023.120075)
Supplement: 1 [file NIHMS1896482-supplement-1.docx]

**Supplementary Table 1.** T1-weighted image parameters from the 8 study sites.

| Site | Manufacturer | Field  Strength  (T) | Image  Dimension  (mm) | Slice  Thickness  (mm) | TR  (msec) | TE  (msec) | Flip  Angle  (deg) |
| --- | --- | --- | --- | --- | --- | --- | --- |
| 1 | Siemens | 1.5 | 256 x 256 x 160 | 1.60 | 25.00 | 4.60 | 30 |
| 2 | Siemens | 3.0 | 176 x 240 x 256 | 0.90 | 2250.00 | 3.96 | 9 |
| 3 | Siemens | 3.0 | 128 x 256 x 256 | 1.33 | 6.60 | 2.90 | 8 |
| 4a | GE | 1.5 | 124 x 256 x 256 | 1.2 | 11.10 | 2.20 | 25 |
| 4b | GE | 1.5 | 124 x 256 x 256 | 1.40 | 11.10 | 2.20 | 25 |
|  |  |  |  |  |  |  |  |
| 5 | Siemens | 3.0 | 160x 256 x 256 | 1.00 | 1600.00 | 3.37 | 15 |
| 6 | Philips | 1.5 | 170 x 256 x 256 | 1.00 | 8.02 | 3.69 | 7 |
| 7 | GE | 1.5 | 181 x 217 x 181 | 1.00 | 6.00 | 63.00 | -- |
| 8 | Siemens | 1.5 | 160 x 256 x 256 | 1.00 | 2000 | 3.65 | 8 |

The imaging parameters above were obtained from de-identified DICOM information when available, but from related manuscripts or image header information, which is why flip angle information is missing for data from one site. The relatively long TRs above were used for inversion recovery acquisitions (inversion times: site 2, 900 ms and site 5, 640 ms). 4a,b- Data were obtained for 2 different studies from a research site.
